# A single *Proteus mirabilis* lineage from human and animal sources: a hidden reservoir of OXA-23 or OXA-58 carbapenemases in Enterobacterales

**DOI:** 10.1038/s41598-020-66161-z

**Published:** 2020-06-08

**Authors:** Rémy A. Bonnin, Delphine Girlich, Agnès B. Jousset, Lauraine Gauthier, Gaëlle Cuzon, Pierre Bogaerts, Marisa Haenni, Jean-Yves Madec, Elodie Couvé-Deacon, Olivier Barraud, Nicolas Fortineau, Philippe Glaser, Youri Glupczynski, Laurent Dortet, Thierry Naas

**Affiliations:** 1 UMR 1184, Team Resist, INSERM, Paris-Saclay University, Faculty of Medicine, Le Kremlin-Bicêtre, France; 2 French National Reference Center for Antibiotic Resistance: Carbapenemase producing Enterobacteriaceae, Le Kremlin-Bicêtre, France; 3 Joint research Unit EERA « Evolution and Ecology of Resistance to Antibiotics », Institut Pasteur-APHP-University Paris Sud, Paris, France; 4 Bacteriology-Hygiene unit, Assistance Publique - Hôpitaux de Paris, Bicêtre Hospital, Le Kremlin-Bicêtre, France; 5 Belgian National Reference Laboratory for Monitoring of Antimicrobial Resistance in Gram-Negative Bacteria, CHU UCL Namur, B-5530 Yvoir, Belgium; 6 Unité Antibiorésistance et Virulence Bactériennes, Université de Lyon - ANSES Laboratoire de Lyon, 31 avenue Tony Garnier, 69364 Lyon, France; 7 Université de Limoges, INSERM, CHU Limoges, UMR 1092 Limoges, France

**Keywords:** Antimicrobial resistance, Clinical microbiology

## Abstract

In Enterobacterales, the most common carbapenemases are Ambler’s class A (KPC-like), class B (NDM-, VIM- or IMP-like) or class D (OXA-48-like) enzymes. This study describes the characterization of twenty-four OXA-23 or OXA-58 producing-*Proteus mirabilis* isolates recovered from human and veterinary samples from France and Belgium. Twenty-two *P. mirabilis* isolates producing either OXA-23 (n = 21) or OXA-58 (n = 1), collected between 2013 and 2018, as well as 2 reference strains isolated in 1996 and 2015 were fully sequenced. Phylogenetic analysis revealed that 22 of the 24 isolates, including the isolate from 1996, belonged to a single lineage that has disseminated in humans and animals over a long period of time. The *bla*_OXA-23_ gene was located on the chromosome and was part of a composite transposon, Tn*6703*, bracketed by two copies of IS*15∆II*. Sequencing using Pacbio long read technology of OXA-23-producing *P. mirabilis* VAC allowed the assembly of a 55.5-kb structure encompassing the *bla*_OXA-23_ gene in that isolate. By contrast to the *bla*_OXA-23_ genes, the *bla*_OXA-58_ gene of *P. mirabilis* CNR20130297 was identified on a 6-kb plasmid. The acquisition of the *bla*_OXA-58_ gene on this plasmid involved XerC-XerD recombinases. Our results suggest that a major clone of OXA-23-producing *P. mirabilis* is circulating in France and Belgium since 1996.

## Introduction

*Proteus* spp. are Gram-negative rods and belong to the order of Enterobacterales and to the family of *Morganellaceae*. This genus is part of the natural gut microbiota in humans and animals. Six species compose this genus being *Proteus mirabilis, Proteus vulgaris, Proteus penneri, Proteus cibarius, Proteus terrae* and *Proteus hauseri*, and three genomospecies 4, 5, and 6^1,2^. Among these species, *P. mirabilis* is the most commonly identified from clinical samples, mainly in context of urinary tract infections (UTIs) but also from a wide range of clinical samples related to healthcare associated infections^3^. *P. mirabilis* does not produce any intrinsic β-lactamase. Accordingly, the wild-type resistance pattern is fully susceptible to all β-lactams active on Enterobacterales. Resistance to cephalosporins in *P. mirabilis* is caused by the acquisition of extended-spectrum β-lactamases (ESBLs) of CTX-M-, VEB- and PER-types or of plasmid-mediated cephalosporinases such as CMY-type^2,4–6^. Some of the most prevalent carbapenemases in Enterobacterales were sporadically described in *P. mirabilis* isolates including KPC-2, VIM-1, IMP-like, NDM-1 and OXA-48^7–9^.

The carbapenem-hydrolyzing class D β-lactamases (CHDLs) of *Acinetobacter* spp. are divided into five phylogenetic distinct subgroups: OXA-23-like, OXA-24/-40-like, OXA-51-like, OXA-58-like and OXA-143^10^. As opposed to Enterobacterales other than *P. mirabilis*, the three most prevalent acquired carbapenemases identified in *Acinetobacter* spp. (being OXA-23, OXA-24 and OXA-58) have also been described in *P. mirabilis*: OXA-24/-40 in Algeria, OXA-58 in Belgium and Germany and OXA-23 in France and Finland^11–15^.

The aim of this study was to characterize at the genomic level a collection of OXA-23- and OXA-58-producing *P. mirabilis* isolates recovered from human and animal sources from France and Belgium.

## Results

**Figure 1:**
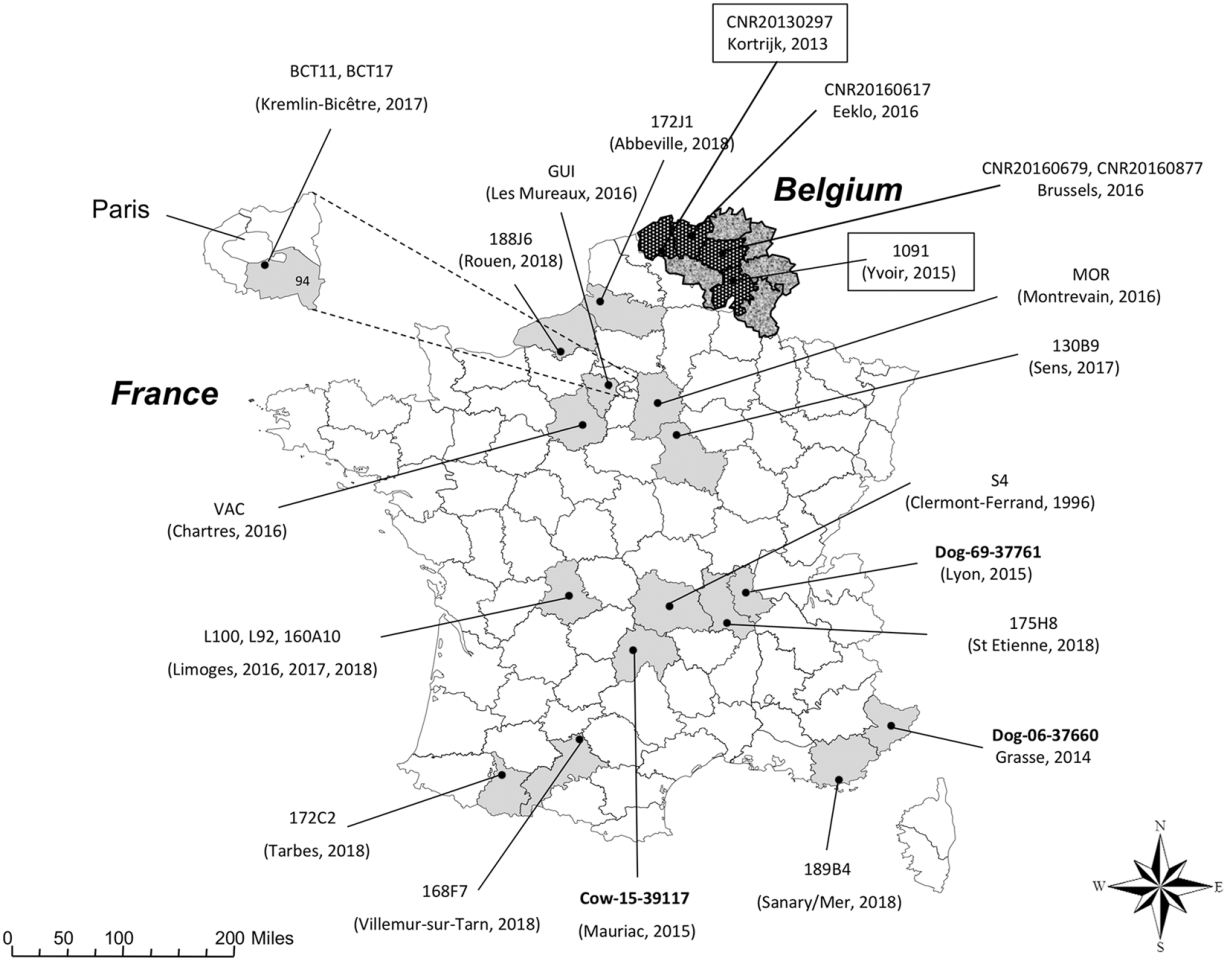
Geographic distribution of the 24 OXA-23- or OXA-58-producing *P. mirabilis* isolates. Bolded isolates correspond to animal isolates, while OXA-58-producing isolates were boxed.

A collection of 61 isolates with phenotypes compatible with the production of OXA-23 or OXA-58 was tested using the lateral flow immunochromatographic assay NG-test Carba 5 (NG Biotech, Guipry, France) test, Carba NP test and PCRs. None of the common enterobacterial carbapenemases (OXA-48-like, NDM, KPC, VIM, and IMP) were detected. Nevertheless, among the 61 isolates, 21 were positive for a *bla*_OXA-23_ gene and one for a *bla*_OXA-58_ gene. These isolates originated from many different areas in France and Belgium (Table 1 & Fig. 1) and were collected over a 4-years period. OXA-23, and OXA-58 CHDLs are weak carbapenem-hydrolyzing enzymes. When they are expressed in *E. coli*, they confer a slightly reduced susceptibility to carbapenems. Recently, we have identified the first OXA-58-producing *P. mirabilis* clinical isolate 1091^11^, that was resistant to amoxicillin, ticarcillin and clavulanate-amoxicillin combination. This strain also showed a reduced susceptibility to ertapenem with MIC over the EUCAST screening cut-off for carbapenemase-producing Enterobacterales (CPE) (>0.125 μg/ml or diameter inhibition zone size <25 mm). Resistance phenotypes of all OXA-producing *P. mirabilis* are summarized in Table S1. A similar pattern was observed for all isolates with a antibiotic susceptibility pattern of clavulanate-amoxicillin resistance and decreased susceptibility to carbapenem. They were all susceptible to broad-spectrum cephalosporins, fluoroquinolones, tigecycline, fosfomycin and amikacin. Few differences were observed on gentamicin and tobramycin, with few isolates being susceptible to these compounds whereas the others were resistant to both of them.

**Table 1:** Clinical features of OXA-23 or OXA-58-producing *P. mirabilis* isolates.

Isolates	Source, sample	Carbapenemase	City, Country (F/B)^a^	Year of isolation	Reference	GenBank accession numbers
1091	Human, blood	OXA-58	Yvoir, B	2015	^10^	MCOR00000000
CNR20130297	Human, N/A	OXA-58	Kortrijk, B	2013	This study	SPTE00000000
S4	Human, N/A^b^	OXA-23	Clermont-Ferrand, F	1996	^16^	SPTF00000000
Cow-15-39117	Cow, blood	OXA-23	Mauriac, F	2015	This study	SPTD00000000
Dog-06-37660	Dog, otitis	OXA-23	Grasse, F	2014	This study	SPTC00000000
Dog-35-37761	Dog, otitis	OXA-23	Lyon, F	2015	This study	SPTB00000000
L100	Human, skin biopsy	OXA-23	Limoges, F	2016	This study	SPTA00000000
L92	Human, stool	OXA-23	Limoges, F	2016	This study	SPSZ00000000
CNR20160679	Human, Respiratory	OXA-23	Brussels, B	2016	This study	SPSY00000000
CNR20160877	Human, Urine	OXA-23	Brussels, B	2016	This study	SPSX00000000
CNR20160617	Human, Urine	OXA-23	Eeklo, B	2016	This study	SPSW00000000
GUI	Human, Urine	OXA-23	Les Mureaux, F	2016	This study	SPSV00000000
VAC	Human, Rectal swab	OXA-23	Chartres, F	2016	This study	CP042907
MOR	Human, Urine	OXA-23	Montevrain, F	2016	This study	SPST00000000
BCT11	Human, Urine	OXA-23	Le Kremlin-Bicetre, F	2017	This study	SPSS00000000
BCT17	Human, Urine	OXA-23	Le Kremlin-Bicetre, F	2017	This study	SPSR00000000
130B9	Human, Urine	OXA-23	Sens, F	2017	This study	SPTN00000000
160A10	Human, Urine	OXA-23	Limoges, F	2018	This study	SPTM00000000
168F7	Human, Urine	OXA-23	Villemur sur Tarn, F	2018	This study	SPTL00000000
172C2	Human, Blood	OXA-23	Tarbes, F	2018	This study	SPTK00000000
172J1	Human, Urine	OXA-23	Abbeville, F	2018	This study	SPTJ00000000
175H8	Human, Urine	OXA-23	Saint-Etienne, F	2018	This study	SPTI00000000
188J6	Human, Urine	OXA-23	Rouen, F	2018	This study	SPTH00000000
189B4	Human, N/A	OXA-23	Sanary/Mer, F	2018	This study	SPTG00000000

^a^F: France, and B: Belgium ^b^. N/A: not available.

### Resistome of OXA-23-/OXA-58-producing *P. mirabilis* isolates

**Figure 2:**
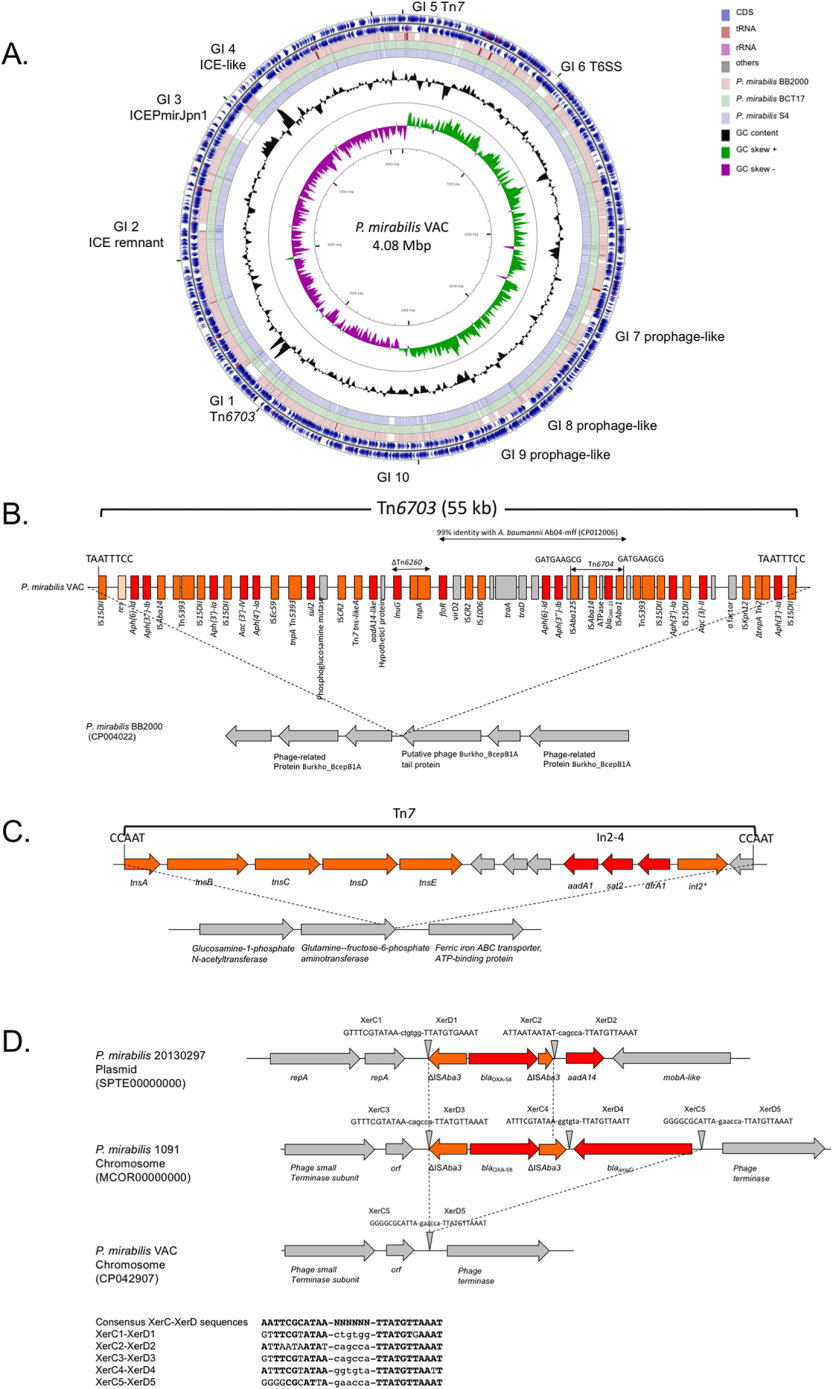
(**A**) Comparative genomic of *P. mirabilis* VAC. Genome analysis of *P. mirabilis* VAC and its comparison with *P. mirabilis* S4 (1996), *P. mirabilis* BCT17 (2017) and *P. mirabilis* BB2000 reference genome (CP004022). Circular representation was obtained using CGViewer. Inner circles represent CG content (black circle) and CG Skew (green & purple circle). GI = Genomic Island **(B)** Schematic representation of IS*15∆II*-based composite transposon (Tn*6703*) and its insertion site. Red boxes represent resistance genes and orange boxes represent mobile elements. **(C**) Schematic representation of Tn*7*. Genes are indicated by arrows. Red arrows represent resistance genes and orange arrows represented mobile elements. **(D)** Analysis of the genetic context of *bla*_OXA-58_ in *P. mirabilis* 1091 and 20130297 isolates. XerC-XerD binding sites are indicated by triangles. Dashed lines represent DNA insertions.

WGS of all the OXA-23- and OXA-58-producing *P. mirabilis* isolates (n = 22) of this study along with OXA-58-producing *P. mirabilis* 1091, and OXA-23-producing *P. mirabilis* S4^11,16^ were performed using Illumina technology. Resistomes were determined using the Resfinder 3.1 and the CARD database^17,18^. They are summarized in Table S2. The *P. mirabilis* VAC possessed the highest number of acquired resistance determinants. It carried multiple aminoglycoside resistance genes (two copies of *aph(6′)-Id*, three copies of *aph(3″)-Ib*, *aac(3)-IV*, *aph(4)-Ia*, *aadA1*, *aadA14-like*, two copies of *aph(3’)-Ia* and *aac(3’)-II*), the phenicol resistance gene *floR*, the lincosamide nucleotidyltransferase gene *lnuG*, the sulfonamide resistance gene *sul2*, a streptothricin acetyltransferase gene *sat2*, and the carbapenem resistance *bla*_OXA-23_ gene (Table S2). Accordingly, this strain was selected as reference for further analyses and sequenced using the PacBio technology. Sequencing gave 27,741 reads representing a total of 166 521,740 nucleotides. The genome of *P. mirabilis* VAC was reconstructed and was 4.08 Mb in size with a GC content of 39% (Fig. 2A).

### Phylogenetic analysis of *bla*_OXA_-containing *P. mirabilis* isolates

**Figure 3:**
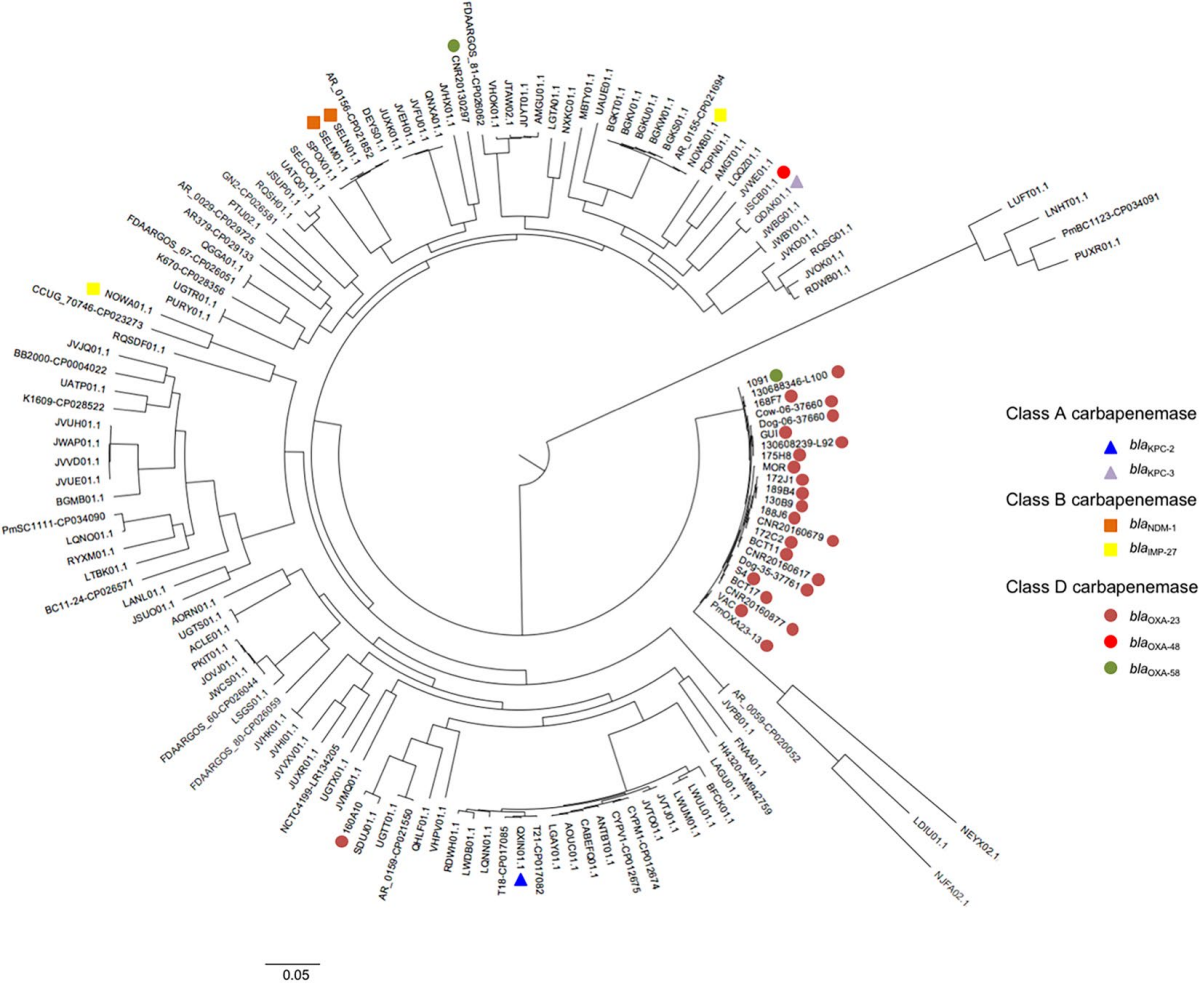
Phylogenetic relationship of the 24 OXA-23 and OXA-58-producing *P. mirabilis* isolates with 121 reference genomes of *P. mirabilis* from GenBank. The phylogenetic tree was obtained using CSI phylogeny v1.4^43^. Carbapenemase producing isolates are labelled with their respective coloured symbols.

To dive deeper into the understanding of the dissemination of the *bla*_OXA-23_ or *bla*_OXA-58_ genes, the genome sequences of all sequenced *P. mirabilis* isolates were compared. In addition, 122 available reference genomes of *P. mirabilis* from GenBank were also included in the analysis (Table S2). Surprisingly, 22 of the 24 CHDL-producing isolates, including the OXA-23-producing *P. mirabilis* S4 and the OXA-58-producing *P. mirabilis* 1091, belonged to the same lineage (Fig. 3). Single nucleotide polymorphisms (SNPs) count revealed that 22 isolates possessed the same background (less than 50 SNPs *vs* > 2000 SNPs for unrelated clones) confirming that all these isolates belonged to the same lineage. Moreover, despite the fact that three isolates (NEYX, NJFA and LDIU) were branched to OXA-producing lineage, they are not related with an average of 4,200, 4,900 and 5,000 SNPs respectively with the OXA-23/OXA-58-producing isolates (Fig. 3 and Table S3). Two OXA-producing isolates (*P. mirabilis* 160A10 and CNR20130297) were not related to the main lineage (>2000 SNPs). Isolate 160A10 and SDUJ01 are close with 185 SNPs (Table S3) whereas isolate CNR20130297 was a singleton.

In addition to the carbapenemase-encoding gene (*bla*_OXA-23_ or *bla*_OXA-58_), all isolates from the main cluster carried acquired aminoglycoside and sulfonamide resistance genes (Table S2). The unrelated OXA-58-producing *P. mirabilis* CNR20130297 and OXA-23-producing *P. mirabilis* 160A10 displayed different resistance features. As opposed to the isolates of the main cluster, *P. mirabilis* CNR20130297 remained susceptible to all tested aminoglycosides (gentamicin, tobramycin, kanamycin, amikacin and netilmicin), and both isolates (CNR20130297 and 160A10) produced an additional β-lactamase TEM-1 (Table S2).

Of note, a chloramphenicol acetyltransferase gene (*cat*) and a tetracycline efflux pump encoding gene (*tet(J)*), both related to the intrinsic resistance to tetracyclines and chloramphenicol of *P. mirabilis* species were present in all genomes.

### The *bla*_OXA-23_ gene is carried by a transposon on the chromosome

Attempts to transfer the *bla*_OXA-23_ carbapenemase gene from *P. mirabilis* VAC by conjugation and transformation failed. Genome analysis using *P. mirabilis* VAC as reference for the dominant OXA-23-producing clone (see above) confirmed that the *bla*_OXA-23_ gene was located on the chromosome. Comparative genomics between the *P. mirabilis* VAC isolate and the fully susceptible *P. mirabilis* BB2000 reference strain revealed the presence of genomic islands (GIs) only in the *P. mirabilis* VAC isolate (Fig. 2A). Here, GIs refer to large DNA sequences coming from an horizontal transfer and integrated in the chromosome^19^. Among these GIs, GI1 corresponds to the Tn*6703* transposon that carries the *bla*_OXA-23_ gene. GI3 shares 97% of nucleotide identity with an integrative and conjugative elements (ICE) ICEPmiJpn1 identified in *P. mirabilis* (KY437729). GI4 another ICE identified in different *P. mirabilis* isolates as well as in *Klebsiella quasipneumoniae* strain KPC142 (CP023478), *Providencia stuartii* strain BE2467 (CP017054) and *Morganella morganii* strain AR_0133 (CP028956). GI5 is a copy of the class 2 transposon Tn*7*. Finally, GI6 contains a putative type VI secretion system encoding operon.

In *P. mirabilis* VAC, the *bla*_OXA-23_ gene is carried on GI1 of 55-kb in size. It is bracketed by two copies of IS*15∆II*, an IS*26* point mutant variant belonging to the IS*6* family^20^. IS*15∆II* themselves are bracketed by a target site duplication (TSD) TAATTTCC (Fig. 2B), typical of IS*15∆II* (as well as IS*26*) transposition events^21–23^. This composite transposon was named Tn*6703* according to the transposon registry database (https://transposon.lstmed.ac.uk/). It has been previously demonstrated that at least 6 copies of *bla*_OXA-58_ gene were duplicated in tandem in *P. mirabilis* 1091^11^. Conversely to what was reported in *P. mirabilis* 1091 isolate, only one copy of the *bla*_OXA-23_ gene was present in all isolates of the main clone (ratio *bla*_OXA-23_/housekeeping genes at 1). Analysis of the close genetic structure of *bla*_OXA-23_ gene revealed that it was carried by a Tn*2008*-like transposon named Tn*6704* (Fig. S1)^24^. Tn*6704* was inserted in a fragment of Tn*5393* within a non-coding region between the resolvase and *strA* genes. Noticeably, a plasmid replicase from *Acinetobacter* was identified within this Tn*6704*. However, this replicase encoding gene is interrupted by IS*Aba125* (bracketed by TSD of 3 bp, TAG). This Tn*6704* is, itself, bracketed by TSD of 9-bp (GATGAAGCG) consistent with IS*Aba1*-based transposition (Figs. 2B and S1). Alignment of IR of IS*Aba1* and the putative IRL found at the left extremity of Tn*6704* revealed a weak nucleotide sequence identity with IRL of IS*Aba1* (Fig. S1). As usually reported, IS*Aba1* is present upstream of *bla*_OXA-23_ gene in Tn*6704*. IS*Aba1* is known to be involved in *bla*_OXA-23_ gene expression^25^. Downstream of the *bla*_OXA-23_ gene, an ATPase-encoding gene was identified, as described in all transposons carrying *bla*_OXA-23_^10^_._ Following this ATPase-encoding gene, a copy of IS*Aba14* and of IS*Aba125* were identified. Downstream the Tn*6704*, a IS*15∆II*-mediated putative transposon carrying *aph(3′)-Ia* is present, followed by *aac(3′)-II* genes. Then, a putative sigma factor sharing homology with σ factor from environmental bacteria was identified (52% ID AA WP_127199220), followed by a copy of IS*Kpn12*, an *aph(3′)-Ia* gene and another IS*15∆II* copy (Fig. 2B).

A region covering ca. 20% of the GI1, including the Tn*6704*, shares 99% of nucleotide identity with *A. baumannii* genome Ab04-mff (CP012006) (Fig. 2B.). This region contained a copy of IS*Aba125* followed by two aminoglycoside resistance genes (*aph(6′)-Id* and *aph(3′)-Ib*) and two genes involved in plasmid transfer (*traA*/*traD*). This structure was identified in all OXA-23-producing *P. mirabilis* of this study. Intriguingly, close to this region and only in *P. mirabilis* VAC, a fragment of Tn*6260* carrying *lnu(G)* resistance gene originating from *Enterococcus faecalis* was identified^26^. The *lnu(G)* gene was bracketed by two copies of IS*CR2*, an IS*91*-like mobile element. Ultimately, two copies of IS*15∆II* bracketed this MDR GI with a TSD of 8-bp (TAATTTCC) leading to a putative composite transposon. Of note, all isolates of this clone do not share the same resistome (Fig. 2A.). The alignment of whole genome sequences revealed some differences in this region. This can be explained, for instance, by the presence of the transposon carrying *lnuG* only in *P. mirabilis* VAC. Accordingly, this *lnuG*-carrying transposon was most likely acquired recently. Some aminoglycoside resistance genes are also present in few isolates. The genetic diversity of Tn*6703* is not surprising since studied isolates were recovered from different countries, over a long period and from animal or human. They were likely submitted to different selective pressures that might explain this diversity.

In *P. mirabilis* VAC and other isolates of the same clone, GI1 was inserted within the remnant (15 kb in size) of a prophage sharing 75% nucleotide identity with a prophage identified in *Providencia rettgeri* RB151 (CP017671). *P. mirabilis* BB2000 reference strain (CP004022) also harboured this prophage, but neither Tn*6703* nor any resistance genes were inserted in it (Fig. 2B.). In the unrelated *P. mirabilis* 160A10, the *bla*_OXA-23_ gene was also part of a Tn*6703*-like element. However, since 160A10 possessed an intact homolog of the phage Burkho_BcepB1A tail protein-encoding gene (GenBank NC005886). To decipher the genetic context of the carbapenemase gene in this isolate, *P. mirabilis* 160A10 was sequenced using MinIon technology. In this isolate, the *bla*_OXA-23_ gene is carried by a conjugative plasmid of 67 kb in size (Fig. S2). This plasmid carried a full transfer operon and was not typeable using PlasmidFinder v2.1 for replicon typing of Enterobacterales. The *bla*_OXA-23_ gene was present within a fragment of Tn*6703* carried by the plasmid (Fig. S2)

The other resistance genes (*aadA1*, *sat2* and *dfrA1*) were identified within a class 2 integron carried by a Tn*7* transposon (GI5) (Fig. 2C.). This transposon has been identified in many isolates of *P. mirabilis*^27^. As previously reported, the class 2 integrase gene contains a premature stop codon leading to a pseudo-gene (Fig. 2C.)^28^.

### The *bla*_OXA-58_ gene might be mobilized by XerC/XerD recombination events

Within *P. mirabilis* CNR20130297, the *bla*_OXA-58_ gene is carried on a plasmid of 6,219 bp that shared 99,9% nucleotide identity (only one SNP), with plasmid p10797-OXA-58 (KU871396). This plasmid has been previously identified in a OXA-58-producing *P. mirabilis* from Germany^12^. The plasmid replicase showed 51% amino acid identity with a replicase of *Stenotrophomonas maltophilia* (GenBank accession number WP_029214130.1) and to a lesser extent with another replicase of *Acinetobacter lwoffii* (50% amino acid identity) (GenBank accession number WP_005102557.1). Analysis of the closed genetic environment of the *bla*_OXA-58_ gene revealed that XerC-XerD recombination was likely involved in its acquisition (Fig. 2D.). The process of site-specific recombination can be performed by two chromosomally-encoded tyrosine recombinases (XerC and XerD). These recombinases recognize a 28-bp recombination site named *dif* and may allow resolution of the recombination event^29^. XerC and XerD recombination sites are composed of two sequences of 11 nucleotides separated by a spacer of 6 nucleotides^30^. In *P. mirabilis* 1091, the *bla*_OXA-58_ gene was bracketed by two fragments of IS*Aba3*, and a gene coding for a cephalosporinase as previously described^11,31^. Bracketing IS*Aba3*-*bla*_OXA-58_-IS*Aba3*, two XerC-XerD sites were identified named XerC3/XerD3 and XerC4/XerD4. Downstream of the *bla*_ampC_ gene, another site was identified called XerC5/XerD5. In *P. mirabilis* VAC, only XerC5/XerD5 is present and might be considered as an empty XerC-XerD binding site within a prophage (Fig. 2D.). In *P. mirabilis* CNR20130297, harbouring the p20130297-OXA-58 plasmid, XerC1/XerD1 binding site is found at the 5’ end extremity of the structure whereas a XerC2-XerD2 binding site is present at the 3’ end extremity. Analysis of XerC-XerD sites suggests a mobilisation of this structure *via* XerC-XerD recombinases.

## Discussion

OXA-23 is the main carbapenemase identified in *Acinetobacter* species. The *bla*_OXA-23_ gene is now widespread and even endemic in some areas^32^. However, this carbapenemase is very rarely identified in Enterobacterales. Only a few CHDL, other than OXA-48-like carbapenemases, have been reported in Enterobacterales and especially in *Proteus* spp^11–15^.

Here, we report the first genomic characterization of twenty-one OXA-23- and one OXA-58-producing *P. mirabilis* isolates from 2013 to 2018. Two reference OXA-producing *P. mirabilis* isolates were also sequenced: an OXA-23-producer isolated in France in 1996^16^ and the OXA-58-producing *P. mirabilis* 1091 isolated in Yvoir, Belgium, in 2015^11^. This analysis revealed that one clone carrying *bla*_OXA-23_ gene is circulating since 1996 and had spread over the last twenty years among humans and animals. Interestingly, the recently described OXA-58-producing *P. mirabilis* 1091 isolate^11^ also belonged to this lineage (Fig. 3). The comparison with genomes recovered from GenBank revealed that this lineage is distantly related to others lineages except a branch represented by three isolates (NEYX02.1, LDIU01.1 and NJFA02.1). Nevertheless, despite being of the same lineage, these three isolates that do not carry any carbapenemase-encoding gene, are not part of this OXA-23/OXA-58-producing “successful” clone (4000 to 5000 SNPs) (Fig. 3 and Table S3).

The *bla*_OXA-23_ gene is part of a Tn*6704*, which is embedded in a 55-kb DNA sequence bracketed by two copies of IS*15∆II*, thus forming a composite transposon, named Tn*6703*. This transposon is bracketed by an 8-bp target site duplication compatible with an IS*15∆II-*mediated transposition event^21–23^. It is unlikely that this structure was acquired in one step since the mapping of reads on GI1 revealed variability of its content among different isolates. Of note, three resistance genes (*aadA1*, *sat2* and *dfrA1*) were not present in Tn*6703* transposon but carried by Tn*7* (Fig. 2C.). The class 2 integron, carrying these genes, does not seem to be functional anymore. Indeed, the *int2* gene carried a premature stop codon leading to an incomplete integrase. Regarding the *bla*_OXA-58_ gene, its acquisition involved a XerC-XerD tyrosine recombinases and it has been identified either on the chromosome or on a plasmid. XerC-XerD tyrosine recombinases have been involved in the resolution of plasmid co-integrates carrying the *bla*_OXA-58_ gene in *A. baumannii*^33^. Interestingly, this plasmid was reported to replicate in Enterobacterales and in *A. baumannii* ATCC17978^12^. Accordingly, we might hypothesize that this plasmid might be the shuttle vector between the *Acinetobacter* genus and *P. mirabilis*.

Comparative genomics also revealed the presence of other GIs in *P. mirabilis* VAC as compared to the *P. mirabilis* BB2000 reference strain. Among the identified GIs, an ICE sharing high homology with ICEPmiJpn1 (KY437729) has been identified (GI3)^34^. Interestingly this ICE was identified in only two isolates of the main lineage (Fig. 3A.). Several other GIs carrying potential virulence genes were identified in *P. mirabilis* VAC including GI6 carrying a putative type VI secretion system encoding operon. The content of all genomic islands is indicated in Tables S4 and S5. Accordingly, we can speculate that these GIs might be involved in the spread of this clone. Investigations of these elements will be further conducted to decipher their potential role in the spread of this clone.

Here, we described the clonal relationship of OXA-producing *P. mirabilis* over a twenty-one-year period (1996-2017). The spread of the *bla*_OXA-23_ gene is due to a single clone possessing a complex IS*15∆II*-based composite transposon, Tn*6703*. This dissemination could be silent, and the prevalence underestimated since *bla*_OXA-23_ genes are not targeted by most of the carbapenemase detection assays in Enterobacterales. Amplidiag^®^ CarbaR+MCR and CarbaR+MCR (Mobidiag, Paris, France) PCR-based assays are the only commercially-available molecular tests targeting the big 5 carbapenemases (KPC, NDM, VIM, IMP, OXA-48-like), and the main CHDLs from *A. baumannii* (OXA-23, OXA-24/-40, OXA-58, and the over-expressed chromosomally-encoded OXA-51-like β-lactamase associated with an upstream inserted IS*Aba1*). These kits are thus able to detect these carbapenemase producers^35^. Recently, a novel assays either immunochromatographic test targeting OXA-23 in *Acinetobacter* spp., OXA-23 K-SeT^®^ test (Coris BioConcept, Gembloux, Belgium), or molecular assays such as Amplidiag^®^ Carba-R + MCR’s that detects the major carbapenemases: KPC, NDM, VIM, IMP, and OXA-48, as well as the main OXA-type carbapenemases from *Acinetobacter* spp. have been demonstrated to accurately identify OXA-23-producing *P. mirabilis* isolates^35,36^. The use of these assays might help to decipher the underestimated carriage of these OXA-23/58-producing *P. mirabilis*. However, the clinical impact and the need to set-up hygiene measures around these OXA-23/58-producing *P. mirabilis* need to be evaluated since these isolates remain multi-susceptible to most antimicrobials including carbapenems.

## Material and methods

### Strain collection and reference strains

*P. mirabilis* resistant to amoxicillin and amoxicillin-clavulanate sent to the French and Belgium National Reference Centres (NRC) for antibiotic resistance as well as isolates collected through the National Monitoring Network for Antimicrobial Resistance in Diseased Animals (Resapath; https://resapath.anses.fr) were screened for the presence of the *bla*_OXA-23_ or *bla*_OXA-58_ gene. Thus, a total of 61 *P. mirabilis* isolates (4 from the Belgium NRC; 54 from the French NRC and 3 from the Resapath) were collected with a phenotype compatible with the production of a CHDL (Table 1). A collection of 22 OXA-23- and 2 OXA-58- producing *P. mirabilis* were included in this study (Table 1 & Fig. 1). Three isolates were recovered from veterinary samples whereas the others were from human origin. All available reference genomes of *P. mirabilis* from GenBank at the date of November 1^st^ 2019 (n = 122) were used for phylogenetic or comparative genomic analyses.

### Susceptibility testing and carbapenemase detection

Antimicrobial susceptibility testing was performed by the disc diffusion method on Mueller-Hinton (MH) agar (Bio-Rad, Marnes-La-Coquette, France) and interpreted according to EUCAST guidelines (http://www.eucast.org). MICs were determined as recommended using Etest^®^ (bioMérieux, Marcy l’Etoile, France). Carbapenemase detection was performed using the Carba NP test as previously described^37^. The five most prevalent carbapenemase families in Enterobacterales (KPC, NDM, VIM, IMP and OXA-48-like) were also identified by the immunochromatographic assay NG-test Carba5 (NG Biotech, Guipry, France) according to manufacturer’s instructions^31,38^.

### DNA extraction, PCR, and sequencing

Total DNA for Illumina’s sequencing and conventional PCR was extracted from colonies using the Ultraclean Microbial DNA Isolation Kit (MO BIO Laboratories, Ozyme, Saint-Quentin, France) following manufacturer’s instructions. DNA concentration and purity assessments were determined using a Qubit^®^ 2.0 Fluorometer using the dsDNA HS and/or BR assay kit and Nanodrop 2000 (Thermofisher, Saint-Herblain, France). Conventional PCRs were performed as previously described^39^. Main acquired-carbapenemase encoding genes (*bla*_NDM_, *bla*_IMP_, *bla*_VIM_, *bla*_KPC,_ *bla*_OXA-48_, *bla*_OXA-23_, *bla*_OXA-24/40_, *bla*_OXA-58_) in Enterobacterales and *Acinetobacter* spp. were sought by PCR using primers as previously described^10,11^. The DNA library was prepared using the Nextera XT-v2 kit (Illumina, Paris, France) and then run on NextSeq. 500 automated system (Illumina), using a 2 × 100-bp paired-end approach. *P. mirabilis* VAC DNA was sequenced using PacBio’s technology (www.macrogen.com) and used as reference genome. *P. mirabilis* 160A10 was sequenced using MinIon technology as previously described^40^.

### Bioinformatic analysis

*De novo* assembly and read mappings were performed using CLC Genomics Workbench v10.1 (Qiagen, Les Ulis, France). The acquired antimicrobial resistance genes were identified using Resfinder server v3.1 (https://cge.cbs.dtu.dk/services/ResFinder/) and CARD database (https://card.mcmaster.ca)^17,18^. The genome was annotated using the RAST server^41^. Detection of phage was performed using the PHASTER server (www.phaster.ca)^42^. Genomic Island were detected using Island Viewer 4 (http://www.pathogenomics.sfu.ca/islandviewer/). Phylogenetic analysis was performed using CSIPhylogeny v1.4^43^. The parameters used were as follows: minimum distance between SNPs at 10 bp, minimum Z-score at 1.96, and minimum depth at 10X with a relative depth at 10% per position.

The copy number of *bla*_OXA-23_ was assessed to identify a potential gene duplication event as observed for *bla*_OXA-58_. The gene copy number was calculated using the ratio of the coverage of the *bla*_OXA-23_ gene and that of distantly located single copy chromosomal genes (*rpoB*, *dnaA* and *mdh*). Insertion sequences were identified using the ISfinder database^44^.

### Transfer of β-lactam resistance determinants

Plasmids were extracted using Kieser’s method as previously described^45^. Plasmids were extracted using Kieser’s method and subsequently analysed by electrophoresis on a 0.7% agarose gel as previously described^45^, and attempted to be introduced by electroporation into *E. coli* TOP10. Recombinant *E. coli* were selected on TSA supplemented with 50 µg/ml of amoxicillin as previously described^39^. Conjugation assays using *P. mirabilis* isolates as donors and *E. coli* J53 as recipient strains were performed as previously described^46^.

### Ethic statements

No animal or human experiments were performed in this study. All the human isolates were sent anonymously to the NRCs, and none of the authors had access to any identifying information along with the isolates, and that thus ethical approvals and informed consents were not needed.

### Nucleotide sequence accession number

The whole genome sequences generated in the study have been submitted to the GenBank nucleotide sequence database under the accession numbers detailed in Table 1. The nucleotide sequence of the 6-kb plasmid carrying *bla*_OXA-58_ in *P. mirabilis* CNR20130297 was submitted to the GenBank nucleotide sequence database under the accession number MK533136. The genomes of OXA-23- or OXA-58-producing *P. mirabilis* were submitted to GenBank (bioproject number PRJNA521327).

Transparency declarations: L.D. is co-inventor of the Carba NP Test, which patent has been licensed to bioMérieux (La Balmes les Grottes, France).

## Supplementary information

Dataset1.

Dataset2.

Dataset4.

Dataset3.

Dataset5.
