# Supplementary material for: A single Proteus mirabilis lineage from human and animal sources: a hidden reservoir of OXA-23 or OXA-58 carbapenemases in Enterobacterales
Source: Sci Rep. 2020 Jun 8;10:9160. doi: 10.1038/s41598-020-66161-z (PMC7280188; doi:10.1038/s41598-020-66161-z)
Supplement: Supplementary file 1 — Dataset1. [file 41598_2020_66161_MOESM1_ESM.docx]

**Supplementary information**

**A single *Proteus mirabilis* lineage from human and animal sources: a hidden reservoir of OXA-23 or OXA-58 carbapenemases in Enterobacterales**

Rémy A. Bonnin,^1,2,3^ Delphine Girlich,^1,3^ Agnès B. Jousset,^1,2,3,4^ Lauraine Gauthier,^1,2,3,4^ Gaëlle Cuzon,^1,2,3,4^ Pierre Bogaerts,^5^ Marisa Haenni,^6^ Jean-Yves Madec,^6^ Elodie Couvé-Deacon,^7^ Olivier Barraud,^7^ Nicolas Fortineau,^1,3,4^ Philippe Glaser,^4^ Youri Glupczynski,^5^ Laurent Dortet,^1,2,3,4^ Thierry Naas^1,2,3,4^*

^1^ UMR 1184, Team Resist, INSERM, Paris-Saclay University, Faculty of Medicine, Le Kremlin-Bicêtre, France

^2^ French National Reference Center for Antibiotic Resistance: Carbapenemase producing Enterobacteriaceae, Le Kremlin-Bicêtre, France

^3^ Joint research Unit EERA « Evolution and Ecology of Resistance to Antibiotics », Institut Pasteur-APHP-University Paris Sud, Paris, France

^4^ Bacteriology-Hygiene unit, Assistance Publique - Hôpitaux de Paris, Bicêtre Hospital, Le Kremlin-Bicêtre, France

^5^ Belgian National Reference Laboratory for Monitoring of Antimicrobial Resistance in Gram-Negative Bacteria, CHU UCL Namur, B-5530 Yvoir, Belgium

^6^ Unité Antibiorésistance et Virulence Bactériennes, Université de Lyon - ANSES Laboratoire de Lyon, 31 avenue Tony Garnier, 69364 Lyon, France

^7^ Université de Limoges, INSERM, CHU Limoges, UMR 1092, Limoges, France

Supplemental Figures : 2

Supplemental Tables : 5

**Figure S1. A.** Genetic structure of Tn*6704* carrying *bla*_OXA-23_ in *P. mirabilis* VAC and its comparison with Tn*2008*: Genes and their orientations are indicated by arrows. Direct repeats are indicated in bold. **B.** Sequence alignments of inverted repeats. Identical nucleotides are indicated in bold. IRL-like corresponds to the putative IRL found at the left extremities of Tn*6704*. The IRR and IRL corresponded to the IR of IS*Aba1*.

*TnpA*

*bla*_OXA-23_

AAA ATPase

*P. mirabilis* VAC

Tn*6704*

(Tn*2008-like*)

Tn*2008* (*A. baumannii* ANC 4097APRF0100000)

*TnpA*

*bla*_OXA-23_

AAA ATPase

*TnpA*

*TnpA*

**GATGAAGCG**

TAG

TAG

**GATGAAGCG**

IS*Aba1*

IS*Aba14*

IS*Aba125*

IS*Aba1*

**CAATTCAAC**

**CAATTCAAC**

IRL-like AGAAAAG**A**CAAATGA**G**CCAC**C**TCC**G**

IRR TCTGTGA**A**A**TTTGTCGTGTACAGAG**

IRL TTATCTATT**TTTGTCGTGTACAGAG**

Tn*6704*

A.

B.

*ΔrepA*

*ΔrepA*

*res Tn5393*

*strA*

**Figure S2.** Circular representation of pOXA-23-160A10 and its aligment with *P. mirabilis* VAC. This representation was obtained using CGview. CDS were represented by arrows.


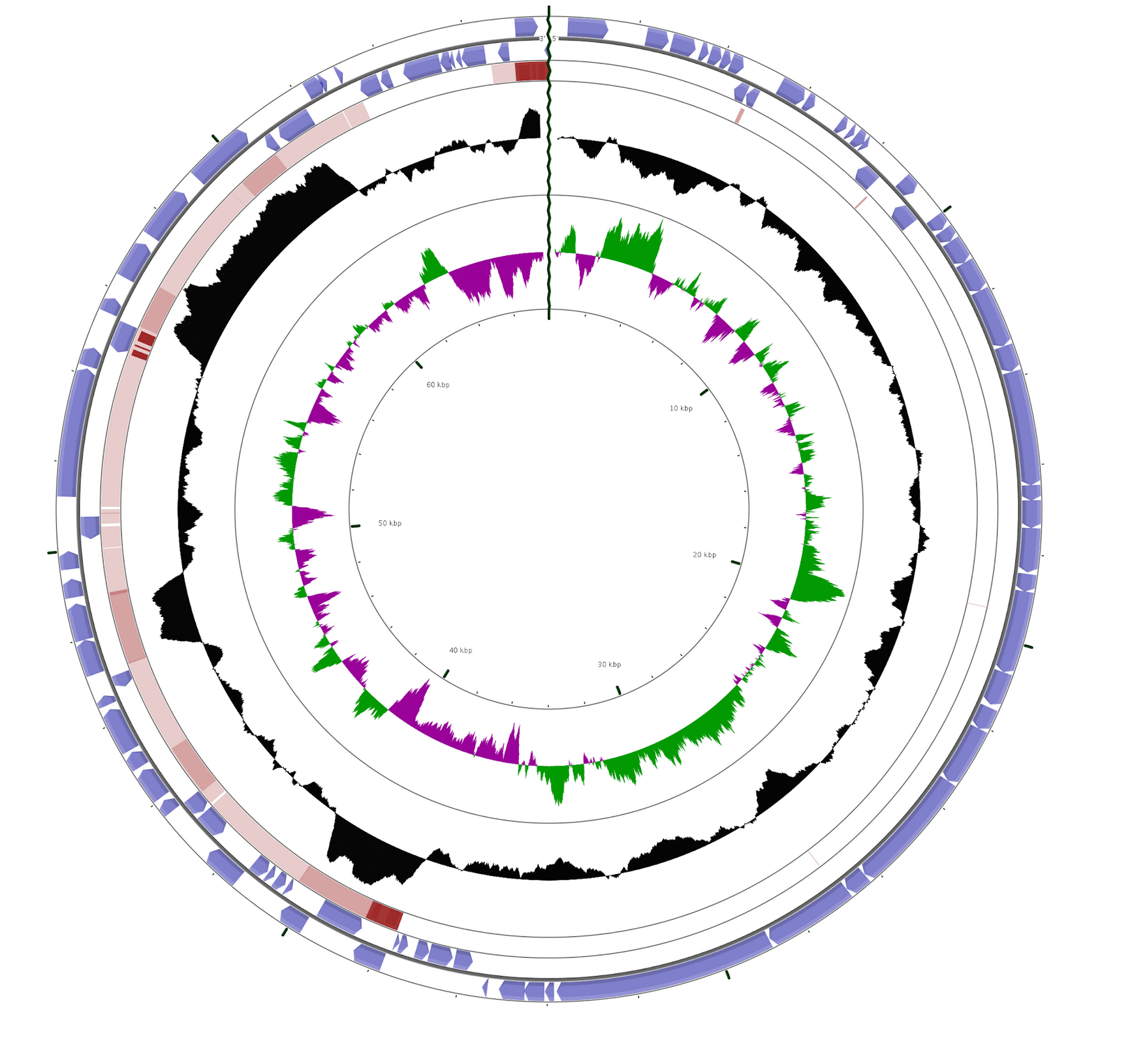

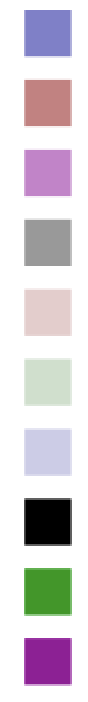


CDS

tRNA

rRNA

Other

Alignment of *P. mirabilis* VAC

GC content

GC Skew +

GC Skew -


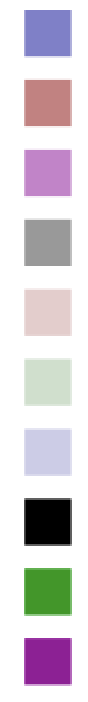


*repA*

*relE/parE* toxin/antitoxin system

*Transfer operon*

*bla*_OXA-23_

IS*26*

IS*26*

IS*Aba2*

IS*Aba1*

IS*Aba14*

IS*Aba125*

pOXA-23-160A10

67924 bp

Homology with Tn*6703*

**Table S1.** Susceptibility testing of OXA-23/OXA-58-producing *P. mirabilis*.

| Isolates | AMX | AMC | TEM | CAZ | ERT | IPM | TE | TGC | CIP | LVX | FOS | SXT | CN | AK | TOB | CT |
| --- | --- | --- | --- | --- | --- | --- | --- | --- | --- | --- | --- | --- | --- | --- | --- | --- |
| 1091 | R | R | R | S | S | S | R | S | S | S | S | R | R | S | R | R |
| CNR20130297 | R | R | S | S | S | S | R | S | S | S | S | R | S | S | S | R |
| S4 | R | R | S | S | S | S | R | S | S | S | S | R | R | S | R | R |
| Cow-15-39117 | R | R | S | S | S | S | R | S | S | S | S | R | R | S | R | R |
| Dog-06-37660 | R | R | S | S | S | S | R | S | S | S | S | R | R | S | R | R |
| Dog-35-37761 | R | R | S | S | S | S | R | S | S | S | S | R | S | S | S | R |
| L100 | R | R | S | S | S | S | R | S | S | S | S | R | R | S | R | R |
| L92 | R | R | S | S | S | S | R | S | S | S | S | R | R | S | R | R |
| CNR20160679 | R | R | S | S | S | S | R | S | S | S | S | R | S | S | S | R |
| CNR20160877 | R | R | S | S | S | S | R | S | S | S | S | R | R | S | R | R |
| CNR20160617 | R | R | S | S | S | S | R | S | S | S | S | R | S | S | S | R |
| GUI | R | R | S | S | S | S | R | S | S | S | S | R | R | S | R | R |
| VAC | R | R | S | S | I | I | R | S | S | S | S | R | R | S | R | R |
| MOR | R | R | S | S | S | S | R | S | S | S | S | R | R | S | R | R |
| BCT11 | R | R | S | S | S | S | R | S | S | S | S | R | S | S | S | R |
| BCT17 | R | R | S | S | S | S | R | S | S | S | S | R | R | S | R | R |
| 130B9 | R | R | S | S | S | S | R | S | S | S | S | R | S | S | S | R |
| 160A10 | R | R | S | S | S | S | R | S | S | S | S | R | R | S | R | R |
| 168F7 | R | R | S | S | S | S | R | S | S | S | S | R | R | S | R | R |
| 172C2 | R | R | S | S | S | S | R | S | S | S | S | R | S | S | S | R |
| 172J1 | R | R | S | S | S | S | R | S | S | S | S | R | R | S | R | R |
| 175H8 | R | R | S | S | S | S | R | S | S | S | S | R | R | S | R | R |
| 188J6 | R | R | S | S | S | S | R | S | S | S | S | R | R | S | R | R |
| 189B4 | R | R | S | S | S | S | R | S | S | S | S | R | R | S | R | R |

**Table S2.** Genomic data of all *Proteus mirabilis* from Genbank.

All isolates from Genbank (1^st^ Septembre 2019) were included in the study. Genbank accession number, genomic informations and resistome are indicated.

**Table S3.** SNP matrix of *P. mirabilis*. SNP count was obtained from CSIphylogeny v1.4 (<https://cge.cbs.dtu.dk/services/CSIPhylogeny/>).

**Table S4.** Genomic Island content of P. mirabilis VAC.Results were obtained using IslandViewer4 (‘http://www.pathogenomics.sfu.ca/islandviewer/).

**Table S5.** Presence of genomic island based on alignment using CGview. “+” indicated the presence of the structure on the genome and “-“ its absence.
